# Are we romanticizing traditional knowledge? A plea for more experimental studies in ethnobiology

**DOI:** 10.1186/s13002-024-00697-6

**Published:** 2024-05-26

**Authors:** Marco Leonti

**Affiliations:** Department of Biomedical Sciences, University of Cagliari, Cittadella Universitaria, 09042 Monserrato, CA Italy

**Keywords:** Experimental studies ethnobiology, Traditional knowledge, Local knowledge, Sustainability, Knowledge dynamics

## Abstract

In answer to the debate question "Is ethnobiology romanticizing traditional practices, posing an urgent need for more experimental studies evaluating local knowledge systems?" I suggest to follow-up on field study results adopting an inclusive research agenda, and challenge descriptive data, theories, and hypotheses by means of experiments. Traditional and local knowledge are generally associated with positive societal values by ethnobiologists and, increasingly also by stakeholders. They are seen as a way for improving local livelihoods, biocultural diversity conservation and for promoting sustainable development. Therefore, it is argued that such knowledge needs to be documented, protected, conserved in situ, and investigated by hypothesis testing. Here I argue that a critical mindset is needed when assessing any kind of knowledge, whether it is modern, local, indigenous, or traditional.

## Introduction

In this essay I take a broad view on ethnobiology highlighting the often-heterogenous origin and fuzzy character of local and traditional knowledge and the importance of being enriched with modern and outside knowledge. As a follow-up on a previous debate about the question of whether ethnobiology should “…abandon more classical folkloric studies” and instead “foster hypothesis-driven forefront research…” I asked for the approval of a call on “are we romanticizing traditional knowledge and is there a need for more experimental studies in ethnobiology” [full stop]. Here I acknowledge the need for descriptive and hypothesis-driven studies but also point out their limitations and the value of experimental studies as a means for overcoming the inherent subjectivity of human observation. Some of the experimental studies I refer to are not strictly ‘ethnobiological’, but they could have been so, if only experimental approaches were more frequently implemented.

Experimentations were instrumental for human cultural progress [1] and are also used for understanding cultural evolution [2]. Experiments are conducted to challenge hypotheses, assess the probabilities of efficacy, or have an exploratory character. It is distinguished between true experiments, where participants or treatments are assigned randomly or quasi-experiments where participants or treatments are selected for groups [3]. The scientific strength of experiments lies in their reproducibility and the consequent logical analysis of the results obtained. In laboratory experiments variables can be controlled for while field experiments are closer to reality [4]. Natural experiments are going on permanently around us and cannot be manipulated by researchers but only evaluated. Since the data is recorded in a natural setting it is crucial to capture the baseline data of the identified variables and to understand which hypothesis is actually being tested [1, 4]. The experiment is an essential part of the scientific progress process [5]. Active involvement in descriptive studies, hypothesis testing and experimental research, grants a more nuanced sense of what evidence is, and insights into the difficulties of human observation.

## What is traditional and local knowledge and what are the dynamics?

Traditional knowledge and customs (referred to also as indigenous and local knowledge) have been reported by natural philosophers and chroniclers since around 2000 years (e.g., [6–8]). With the help of written documents, cultural remains and archaeological artefacts, we understand persistence and dynamics of traditions and knowledge. Besides of being maintained or abandoned, traditional knowledge can synchronize with ‘outside’ knowledge and syncretize, blend with newly generated knowledge, evolve gradually, be reinvented, or invented intentionally [9–11]. For instance, the cheese ‘fondue’, a Swiss national dish, probably now considered traditional by many, was ad hoc invented to promote the consumption of Swiss cheese during the 1930ies and presented to an international audience at the New York World’s Fair 1939/40.

So, what is it that makes knowledge to become ‘traditional’? In the context of herbal medicine, traditional knowledge is defined and distinguished from a current fashion or a trend in that its transmission must involve at least three generations, including two steps of knowledge transmission or, alternatively, three ‘training generations’ where knowledge is being passed on to apprentices [12]. The definition of traditional knowledge in general is fuzzier and many of our daily activities, (e.g., preparing food) contain traditional elements. However, not all activities are traditional just because they have been practiced ever since or because they are sustainable. For instance, collecting rainwater for plant irrigation purposes, is not per se traditional ecological knowledge (TEK). People would collect rainwater for plant irrigation also if they had never observed this practice anywhere else. Moreover, having a theory of mind and observational skills individuals can understand the poor quality and unsustainability of (chlorinated) tap water for watering plants and thus try to avoid the associated economic costs. Collecting rainwater such as roof run-off is just intuitive and logic. On the other hand, complex and elaborate water collection and irrigation systems adapted to specific landscapes and climatic conditions are often grounded on traditional knowledge [13]. It is the culture-specific way of doing things that characterizes the local or the traditional and not necessarily actions per se.

From documents of culture-historical importance we learn that while many traditional practices and customs did not stand the test of time and were wisely abandoned, others persist to date. For instance, many medical treatments were not effective or even dangerous. Bloodletting or purging by means of poisonous botanical drugs with strong emetic and cathartic effects were eventually abandoned along with European humoral medicine [14]. Other botanical drugs have been used continuously throughout the centuries, many of them with acceptable safety profiles [15–19]. However, the European Medicines Agency (EMA) does not confuse generation-long use with efficacy or effectiveness. In absence of clinical data supporting traditional applications the EMA confers the status of ”traditional use” where “sufficient safety data and *plausible* efficacy are demonstrated” (e.g., any application of extracts derived from *Panax ginseng* C.A. Meyer, underground organs) which is different from products with “recognised efficacy” (e.g., application of 20 mg EtOH (60%) extract obtained from *Vitex agnus-castus* L. fruits with a DER of 6-12:1) for premenstrual syndrome.

The tradition of whaling (the hunting of whales, mainly for blubber) was not sustainable and brought several whale species to the brink of extinction which is the reason why whaling got banned in many countries by 1969. Some traditions were given up because of changing moral and ethical standards and by the introduction of new laws. Disputes about the expansion of slavery caused the American Civil War resulting in an official legal ban of slavery in 1865. Many other traditions ignoring individual’s freedom, right to integrity and equality such as female genital mutilation (in many African countries, the Near East and Indonesia), early child marriage (Africa, Near East, the Indian subcontinent, and South-East Asia) or the Indian caste system [20] continue to be practiced while the legacy of Roman law is still present in Western legal thought [21].

### Example of Italy and the economization of TK

Let’s take for example modern Italian culture and economy which are rooted in the country’s rich history and local traditions. Italy shows a marked North–South economic disparity, associated with geography and culture. The varied history of the different Italian regions is reflected in the distinct traditions in food production, cuisine, and craftsmanship. Italy has currently the highest share of elderly people (> 65 years of age) and one of the lowest birth rates within all European countries. This is also conditioned by the late financial independence and economic insecurity of young Italians permitting them to start a family only relatively late in life. This situation poses serious challenges to health care, old age benefits and economy.

Small- and medium-sized enterprises constitute the backbone of the Italian economy with around 75% of all businesses in family hand [22]. The knowledge for securing the highest quality of raw products at the best conditions and the steps, processes, recipes, tools, and machines used during production are well-kept secrets and associated knowledge transmitted only within the family. Since it is easier to collect taxes from a few large businesses than from many small family businesses, Italian tax authorities face more difficulties than other European countries in this regard. Besides that, a low taxpayer commitment, organized crime, corruption, bureaucracy, low productivity due to lack of process innovation [22, 23] are other traditional problems afflicting the Italian economy. Though Italy being a relatively wealthy country the traditional business structure and reliance on local knowledge is also a drawback for economic growth because innovation and modernization occur too often on a relatively low scale which reinforces Italy’s traditional set up.

### Example of Switzerland and TEK

It was recently showcased how TEK is often maintained because of lack of economic resources that would permit the use of more technological equipment and not because of ecological concerns [24]. Also, others (e.g. [25]) concluded, that in the more economically developed regions TEK practices will have a chance to survive only in protected areas where they are used as a tool for biodiversity conservation and where they are fostered by consumers requesting organic and ecologically sustainable food. Topography also plays an important factor in the maintenance of TEK. In mountainous and alpine regions such as the European Alps, TEK and its application is more prominent than in the lowlands, conditioned by the fact that the inclination of the terrain and the marked seasonal changes do not allow for intensive land management and the use of heavy equipment and machinery. This applies also to natural estuaries, river, sea, and lake shores. Agriculture is subsidized all over Switzerland but more heavily in mountainous regions, where otherwise production would not be profitable at all. Switzerland is a wealthy and rich country, which can afford to subsidize agriculture and traditional ways of food production. Thereby, food sovereignty and indigenous food production systems including TEK are maintained at least partially. According to Article 104 of the Federal Constitution, agriculture has a mandate to provide public services. These are each subsidized with a specific type of direct payment. These services include, for example, near-natural, environmentally friendly, and animal-friendly production, the preservation of natural resources and the maintenance of the cultural landscape. In 2022, the federal government paid out a total of around CHF 2.8 billion in direct payments for agriculture [26].

Also, religious denomination can affect land management practices in Switzerland. The sociocultural differences between the protestant canton of Bern and the catholic canton of Lucerne are amongst others reflected in the fact that contrary to the practice followed in the canton of Lucerne, the grassland in the canton of Bern gets cleaned from bitter dock (*Rumex obtusifolius* L.), a noxious weed [27]. In the past also a catholic and a protestant way of tilling the land existed in Switzerland [28]. Historically, the large number of non-working days in the canton of Lucerne and the heavy demands on the population were blamed by numerous travellers and writers for the region’s lagging behind in terms of industrialisation and agricultural development [29].

With the example of Italy, I tried to explain how traditional, and local knowledge can serve as a starting point for innovation, but that success depends on the effective adoption of global knowledge and economic structures. With the example of Switzerland, I tried to highlight that besides economic resources and consumers preferences also topographical particularities, and religion can have a direct influence on the maintenance and practice of TEK. With both, the example of Italy and Switzerland I also tried to highlight the difficulty of defining and identifying ‘systems’ of traditional knowledge. Thus, ‘traditional knowledge systems’ as such can be difficult to grasp because traditional and modern knowledge are often combined and blended in processes that may give way to new traditions. Therefore, here I rather focus on traditional and local knowledge as such and avoid talking about ‘systems’, which are nowadays to be found only in remote areas and isolated civilizations and communities [30].

In summary, history shows, that non-sustainable practices are often abandoned but, also that societal power structures can help to maintain archaic traditions and that traditional and local knowledge, in combinations with purposeful technological experimentation and invention led to innovation which was instrumental for shaping the world and the various human cultures we know today. However, in my view there is nothing wrong or dramatic about abandoning outdated traditions and practices. Here I argue that traditional knowledge should not only not be romanticized [24] but critically questioned, also with the help of experiments, like any other knowledge.

## Are we howling at the moon?

The question as of whether there is an urgent need for more experimental studies evaluating local knowledge systems is related to a previous debate in this series focusing of whether ethnobiology and ethnomedicine should more decisively foster hypothesis-driven forefront research able to turn findings into policy and abandon more classical folkloric studies. I think that there is no need for dramatizing and that the ‘urgency’ is rather related to the question of whether or how proximate ethnobiology is thought to evolve into a branch of multidisciplinary science adopting an inclusive research protocol.

Clearly, for ethnobiology to prosper both, descriptive and hypothesis-driven approaches are needed. Primary data is the basis for all science and descriptive studies fuel hypothesis-driven studies [31–34]. Though I would agree with the statement that well conducted and solid descriptive studies contextualizing and highlighting new data and perspectives are worthier than hypotheses-driven studies pursuing hypotheses for the sake of confirming already known relationships and facts in an anachronistic or non-contextualized way [32, 35].

However, analysing descriptive data for novelty is not simple and requires extensive background knowledge because the tradition of reporting the use of biodiversity by human societies is as old as written history and an immense quantity of recorded data exists. This complicates data accession, handling, and assessment. The inherent difficulty of assessing novelty of ethnomedical survey papers has been noted by Verpoorte in 2008 who proposed the use of a ‘repository’ (database) where the list of plant species and associated data can be integrated systematically, organized in a way that information for specific taxa or uses can be retrieved easily [31]. A brief paper mentioning methodological aspects and providing background data together with a short discussion was suggested to be published along with the database entry. However, this idea has not caught on. A Spanish initiative, however, has realized a reasonable way forward. Besides a national inventory database including traditional knowledge related to biodiversity that is based on descriptive reports, an online interactive platform was created that allows users to submit personal knowledge related to biodiversity and retrieve specific information [36].

While open databases allow for information exchange and their (changing) content for the formulation of hypotheses, Reyes-Garcia has correctly pointed out that results obtained from hypotheses-driven studies do not automatically translate to approved policies or scientific reorientations [33].  Here I must acknowledge that neither do experimental studies. The strengths of ethnobiology and ethnomedicine lie in the possibilities to “draw on theories and methods from the natural sciences, the social sciences, and the humanities” [33]. The flip side of this asset is that the vast breath of ethnobiology potentiates the complexity of seemingly simple research questions augmenting the possibility for overlooking or not being able to account for important confounders. Here lie the benefits of experiments. In experiments variables can be controlled, providing evidence of additional and specific support in favour or against theories and hypotheses. Ethnobiologists often blindly rely on their findings or on the motivation of researchers with different backgrounds and from different disciplines to pick up and draw on their data for experimental research, instead of taking on the challenge themselves and bring their research to the next level. I argue that by including experimental approaches and engaging in translational research next to describing reality and testing hypotheses the contribution of ethnobiology to the Sustainable Development Goals (SDGs) could be more relevant.

Clearly not all hypothesis testing, and experiments are automatically constructive. Lakatos proposed to focus on research programmes instead on isolated hypotheses as the descriptive unit of achievements [5] because research programmes have “auxiliary hypotheses” and a problem-solving machinery serving as a “protecting belt” in place. In the case of interdisciplinary research programmes such as ethnobiology, ethnobotany and ethnopharmacology the protecting disciplines are biology, history, phytochemistry, pharmacology, medicine, cultural anthropology, ecology, agronomy, and economy, including all their methodological and experimental approaches [35].

## Experimental studies in the context of ethnobiology

Probably conditioned by Brent Berlin’s studies (e.g., [37]), for me ethnobiology is closely tied to human interpretation and classification of environmental sensory inputs and the perception of taste, smell, chemesthesis, vision, acoustics, and touch. Berlin and Kay’s research that led to the proposal of basic colour terms as a biological law was based on an experimental approach [38]. The proposed rule about the lexicalization of the colour space associated with stages of linguistic evolution got later relativized by Berlin and Kay themselves and by others [39] but breached a new dimension in ethnobiological research.

It was a natural experiment that lent support to the hypothesis that natural views as opposed to urban sceneries, may have a restorative effect. Patients assigned to a hospital room with a window view on trees were discharged earlier, required less analgesic medication, and suffered from fewer postoperative complications [40]. The positive effect on general health and well-being of practicing Shinrin-yoku (forest bathing) has been assessed by clinical trials [41]. This practice of mindful engagement with sensory stimuli emitted by forest environments originated in Japan. For instance, a comparative study of the physiological and psychological effects of Shinrin-yoku suggests a positive outcome on mental health and blood pressure [42]. A simultaneous contribution of different factors such as physical activity, overall relaxing effect of acoustic signals [43], green environment [44], the pharmacologic effect of plant volatiles and the volatilome [45] are plausible. Music is a universal cultural achievement used in healing rituals and ceremonies [46] and for directing emotions in general (e.g., film industry). Cumulative experimental evidence supports the idea that music has therapeutic potential [47] (especially for improving cognition and memory with patients suffering from dementia and Alzheimer’s disease [48, 49].

In the specific case of forest bathing experiments could help to assess efficacy of individual factors, including their intensity or dose, and their potentiating and synergistic effects lending additional scientific credit to this practice. On the contrary, homeopathy, a more recent alternative and complementary form of medicine that was invented ad hoc has not shown any efficacy beyond the placebo effect, i.e., the meaning response [50, 51]. Evidence-based data is important for informing practitioners, patients, and social security to allow for informed health care choices and health care provision. Clinical studies on the therapeutic efficacy of sensory inputs serves decision making so that, instead of getting prescribed homeopathic medicines or tranquillizers, patients eventually get prescribed a walk in the woods or a combination of different treatments. A meta-analysis including experimental studies found evidence for the efficacy of smell-training on the recovery of olfactory loss [52]. Olfactory loss (anosmia) and taste loss were also frequent symptoms of COVID-19 and found to be the only symptom associated with depressed mood and anxiety following infection [53]. A systematic review based on experimental studies highlighted that in general, depressed patients had increased olfactory dysfunction compared to healthy participants while patients with impaired olfactory performance showed depressive symptoms progressing in severity with increasing olfactory dysfunction [54]. On the other hand, aromatherapy was found to be effective in clinical trials with patients suffering from anxiety and stress [55]. There is thus substantial experimental evidence stemming from a variety of approaches, experiments and perspectives that spending time in nature and the frequent use of aromatic herbs in the treatment of psychological problems in traditional medicine [56–58] has an evidence base. I think this is very nice to know beyond any personal preferences or morbidities.

Also, taste and flavour properties of botanical drugs are often reported to be important selection cues in traditional medicine [14, 59–62]. However, chemosensory qualities in ethnobiological studies are rarely experimentally assessed with the help of double-blind tasting panels and by challenging research participants with samples. Conducting a tasting panel can be fun and provides much more reliable data than simply asking for taste and flavour properties trying to retrieve participants’ memories. In fact, plant drugs can elicit a range of chemosensory perceptions and they do so to varying degrees. Recently we used chemosensory qualities of 700 botanical drugs assessed by 11 panel participants to predict therapeutic uses as described in an ancient medical text. The results, corrected for shared ancestry of botanical species, suggest that chemosensory perception and perceived physiologic effects guided ancient therapeutic knowledge linking it to modern pharmacology albeit aetiologies have completely changed [14]. Experimental evidence also suggests that it is not simply bitter which is the ‘better’ as suggested [62] but that those bitter tasting edible herbs are concomitantly salty or umami in taste which makes them more palatable and acceptable for food [63].

Especially in medicine many new discoveries were made through experimentations, evaluated by means of standardized experiments and due to serendipity in the context of experiments [64, 65]. Without medical and pharmacological experiments most of us would not sit here and read these lines. If the claims made in traditional medicine were all correct there would be no need for ethnopharmacology or evidence-based medicine at all. The panacea would be within anyone’s reach, and we would probably live in a brave new world [66]. However, this is not the case and although indigenous people are generally not waiting for field researchers to poke their nose into community affairs, once accepted as a foreign investigator or collaborator of their medicinal customs, indigenous people are interested in knowing whether their medicines are effective (personal observation). Often, it is impossible to give an informed response because data are not available for all botanical drugs, or they are not meaningful for the traditional context (e.g., antioxidant in vitro effects). This anecdote highlights that also indigenous people may nurture doubts about the efficacy of their medicines and that there exists interest in knowing the other, Western perspective, as well. In this context it is important to distinguish between efficacy which describes the capacity of an agent to produce an effect under standardized conditions and effectiveness, describing the therapeutic success in real-life practice and within a cultural setting [67, 68]. Traditional use can give some indications about safety profiles but adverse effects that manifest with delay such as hepatotoxicity or nephropathy (kidney disease) are not easily recognized. For many indications effectiveness is even more difficult to appreciate because of confounding factors such as severity of symptoms, self-limiting diseases (such as infections) and the restorative power of the human body. Therefore, ethnopharmacologists design laboratory experiments reflecting as accurately as possible the traditional application to provide information about the medicine’s efficacy and possible adverse effects [69, 70].

Importantly, descriptive studies besides informing experimental studies also serve as a basis for meta-analyses and review papers that consider associated experimental studies. Lack of systematic reviews about experimental data providing evidence or its absence regarding traditional and complementary medicine in Mesoamerica and other regions of the world is linked with insufficient health care strategies and culturally pertinent health materials [58]. Integrative medicine is an important pillar for achieving universal health coverage (UHC) for underserved populations and access to appropriate medical care is central for achieving Sustainable Development Goal three (SDG 3 “Ensure healthy lives and promote well-being for all at all ages”; https://sdgs.un.org/goals/goal3) of the UN Agenda 2030 [71, 72]. In order to fill this gap, we used a consensus approach based on the Mesoamerican Medicinal Plant Database to reflect acceptability and therapeutic importance for a critical assessment of the available pharmacological and toxicological data of botanical drugs [58].

The development of medicines for chronic illnesses and life-promoting medications that can be commercialized in the affluent Western world are appreciated more by stakeholders. In urban areas food supplements and remedies for life-style diseases are important product sectors sourced from herbal drugs and plant-based medicines. However, in a situation of health emergency termed double-burden of disease [73] marginalized populations, in addition to combatting insurging life-style diseases, continue to fight neglected infectious diseases with botanical drugs [58, 74]. Investigations of traditional treatments of rare and neglected parasitic and infectious diseases [75–78] deserve more attention by ethnobiologists and ethnopharmacologists because major pharmaceutical companies show little interest in developing medications for a segment of the population with limited purchasing power [79]. Clinical studies involving humans are beyond what single academic groups can do. A possible way to assess the effectiveness of traditional medicines is by conducting retrospective treatment outcome (RTO) studies (open field experiments), where defined disease-related parameters are retrospectively assessed for clinical outcomes [80]. Food drugs qualify as good candidates for RTO studies because they generally show a high acceptance and are associated with low toxicity. Also, the field of veterinary research is accessible for ethnobiologists [81]. For instance, a placebo and antibiotic standard treatment-controlled study assessed the effect of garlic (*Allium sativum* L.) on weight gain and postweaning diarrhoea in piglets. Garlic showed positive effects on weight gain but no prophylactic effect on postweaning diarrhoea leaving the search for anti-diarrhoeal herbal products able to reduce antibiotic treatment open [82].

The therapeutic value of crude animal drugs is often limited to culture-specific symbolism [83]. Belief in therapeutic effectiveness of animal products prompts illegal trade resulting in a negative impact on the probability of survival of wild animal populations as well as the welfare of individual animals [84, 85]. Redirecting therapeutic demand towards products without conservation issues are more likely to be crowned by species conservation success than simply trying to reduce demand without offering alternatives. It was shown that Traditional Chinese Medicine (TCM) users remained unaffected by information appealing to reduced consumption but that especially the more regular TCM users had a positive attitude towards the idea of buying alternative botanical products [86]. In another study, using an online survey directed to 1000 medical practitioners in China, 86% of respondents reported the willingness to substitute animal-based materials with botanical drugs, provided, that safety and effectiveness was comparable [87]. Though it is challenging to find culturally acceptable plant-based substitutes this might be achieved in close collaboration with traditional healers, vendors, and an experimental assessment of consumers preferences. This proposal is not about trumping indigenous peoples’ rights to maintain their traditional health-care practises, but to actively involve them in generating information protecting their environment and traditions.

## Conclusions

While we should avoid transfiguring modern knowledge and science as categorically superior over traditional knowledge there is also no need to romanticize traditional knowledge. Which approaches, strategies and knowledge can provide the best solutions depends always on the specific context. However, for achieving most of the SDGs there is no way around insights gained from experiments. There is much space for ethnobiologists for engaging in experimental research dedicated to sensory biology and health, traditional medicine, ethnopharmacology and ethnoveterinary research, traditional ecological knowledge, domestication of wild edible plant species, food preferences (landraces, wild vegetables and fruits versus commercial crops, evaluation of traditional recipes), pet-therapy (healing with animals), aromatherapy as well as the analysis of music, or incense and smoke constituents in the context or healing rituals. For an impactful ethnobiology, as with most scientific fields, broad interdisciplinary knowledge and a research agenda including next to descriptive, and hypothesis-driven studies also experimental work is essential. Though ethnobiology and TEK have a strong spiritual component, science as practiced today cannot capture it. It is important that ethnobiology avoids spiritual bypassing and that it builds its science on evidence and not on opinions.

## Data Availability

Not applicable.

## Abbreviations

DER: Drug extract ratio
EMA: European medicines agency
EtOH: Ethyl alcohol
SDG: Sustainable development goal
TCM: Traditional Chinese medicine
TEK: Traditional ecological knowledge

## References

[1] Diamond J. Guns, Germs, and Steel. The Fates of Human Societies. New York/London: W.W. Norton and Company; 2005.

[2] Mesoudi A, Whiten A. The multiple roles of cultural transmission experiments in understanding human cultural evolution. Philos Trans R Soc Lond B Biol Sci. 2008;363(1509):3489–501. 18801720 10.1098/rstb.2008.0129 PMC2607337

[3] Shadish WR, Cook TD, Campbell DT. Experimental and quasi-experimental designs for generalized causal inference. Boston: Houghton Mifflin; 2002.

[4] Bernard HR. Research methods in anthropology—qualitative and quantitative approaches. Chapter 5: Research design: experiments and experimental thinking. New York: Altamira Press; 2006.

[5] Lakatos I. The methodology of scientific research programmes. Philosophical papers, vol. 1. Cambridge: Cambridge University Press; 1989.

[6] Pliny. Natural History, Volume I: Books 1–2. Translated by H. Rackham. Loeb Classical Library 330. Cambridge, MA: Harvard University Press; 1938.

[7] Rousseau JJ. Abhandlung über den Ursprung und die Grundlagen der Ungleichheit unter den Menschen. Stuttgart: Reclam; 2012.

[8] Schultes RE, Raffauf RF. The healing forest: medicinal and toxic plants of the northwest Amazonia. Portland: Dioscorides Press; 1990.

[9] Bye R, Linares E, Estrada E. Biological diversity of medicinal plants in Mexico. In: Arnason, et al. (Eds.), Phytochemistry of medicinal plants pp. 65–82. New York: Plenium Press; 1995.

[10] Hobsbawm E, Ranger T. The invention of tradition. Cambridge: Cambridge University Press; 2012.

[11] Richerson PJ, Christiansen MH. Cultural evolution: society, technology, language, and religion (Strüngmann forum reports). Cambridge: MIT Press Ltd; 2013.

[12] Helmstädter A, Staiger C. Traditional use of medicinal agents: a valid source of evidence. Drug Discov Today. 2014;19(1):4–7. 23932953 10.1016/j.drudis.2013.07.016

[13] Baba KM. Irrigation development strategies in sub-Saharan Africa: a comparative study of traditional and modern irrigation systems in Bauchi State of Nigeria. Agric Ecosyst Environ. 1993;45:47–58.

[14] Leonti M, Baker J, Staub P, Casu L, Hawkins J. Taste shaped the use of botanical drugs. Elife. 2024;12:RP90070. 38265283 10.7554/eLife.90070 PMC10945733

[15] Wichtl M. Teedrogen und Phytopharmaka: Ein Handbuch für die Praxis auf wissenschaftlicher Grundlage. 4th ed. Stuttgart: Wissenschaftliche Verlagsgesellschaft; 2002.

[16] Lardos A, Heinrich M. Continuity and change in medicinal plant use: the example of monasteries on Cyprus and historical iatrosophia texts. J Ethnopharmacol. 2013;150(1):202–14. 23994339 10.1016/j.jep.2013.08.026

[17] Dal Cero M, Saller R, Leonti M, Weckerle CS. Trends of medicinal plant use over the last 2000 years in central Europe. Plants. 2022;12(1):135. 36616265 10.3390/plants12010135 PMC9823631

[18] Sõukand R, Kalle R, Prakofjewa J, Sartori M, Pieroni A. The importance of the continuity of practice: ethnobotany of Kihnu island (Estonia) from 1937 to 2021. Plants People Planet. 2024;6:186–96.

[19] EMA, (European Medicines Agency). https://www.ema.europa.eu/en/search?search_api_fulltext=Committee%20on%20Herbal%20Medicinal%20Products%20%28HMPC%29; 2024.

[20] Desai S, Dubey A. Caste in 21st Century India: competing narratives. Econ Polit Wkly. 2012;46(11):40–9. 22736803 PMC3379882

[21] Wieacker F. The importance of roman law for western civilization and western legal thought. 4BC Int’l & Comp L Rev. 1981;4:257.

[22] Glover S, Gibson K. “Made in Italy”; how culture and history has shaped modern Italian business environment, political landscape, and professional organizations. J Bus Divers. 2017;17(1):21–8.

[23] Parisi ML, Schiantarelli F, Sembenelli A. Productivity, innovation and R&D: micro evidence for Italy. Eur Econ Rev. 2006;50(8):2037–61.

[24] Hartel T, Fischer J, Shumi G, Apollinaire W. The traditional ecological knowledge conundrum. Trends Ecol Evol. 2023;38(3):211–4. 36669935 10.1016/j.tree.2022.12.004

[25] Gómez-Baggethun E, Mingorría S, Reyes-García V, Calvet L, Montes C. Traditional ecological knowledge trends in the transition to a market economy: empirical study in the Doñana natural areas. Conserv Biol. 2010;24(3):721–9. 20067484 10.1111/j.1523-1739.2009.01401.x

[26] Agrarbericht. https://agrarbericht.ch/de/politik/direktzahlungen/finanzielle-mittel-fuer-direktzahlungen; 2023.

[27] Poncet A, Schunko C, Vogl CR, Weckerle CS. Local plant knowledge and its variation among farmer’s families in the Napf region, Switzerland. J Ethnobiol Ethnomed. 2021;17(1):53. 34479597 10.1186/s13002-021-00478-5 PMC8414871

[28] Weiss R. Volkskunde der Schweiz. Grundriss, pp. 310. 2. Auflage, Erlenbach-Zürich: Rentsch-Verlag; 1978.

[29] Bucher S. Bevölkerung und Wirtschaft des Amtes Entlebuch im 18. Jahrhundert. Eine Regional-studie zur Sozial- und Wirtschaftsgeschichte der Schweiz im Ancien Régime. Luzerner Historische Veröffentlichungen, Band 1. p 250. Luzern: Rex Verlag; 1974.

[30] Fernández-Llamazares Á, Lepofsky D, Armstrong CG, Brondizio ES, Gavin MC, Lertzman K, Lyver POB, Nicholas GP, et al. Scientists’ warning to humanity on threats to indigenous and local knowledge systems. J Ethnobiol. 2021;41(2):144–69.

[31] Verpoorte R. Primary data are the basis of all science! J Ethnopharmacol. 2012;139(3):683–4. 22020308 10.1016/j.jep.2011.09.054

[32] Łuczaj Ł. Descriptive ethnobotanical studies are needed for the rescue operation of documenting traditional knowledge. J Ethnobiol Ethnomed. 2023;19(1):37. 37679801 10.1186/s13002-023-00604-5 PMC10486101

[33] Reyes-García V. Beyond artificial academic debates: for a diverse, inclusive, and impactful ethnobiology and ethnomedicine. J Ethnobiol Ethnomed. 2023;19(1):36. 37679793 10.1186/s13002-023-00611-6 PMC10486112

[34] Albuquerque UP, Nóbrega Alves RRD. Integrating depth and rigor in ethnobiological and ethnomedical research. J Ethnobiol Ethnomed. 2024;20(1):6. 38183108 10.1186/s13002-023-00643-y PMC10768275

[35] Leonti M, Casu L, de Oliveira Martins DT, Rodrigues E, Benítez G. Ecological theories and major hypotheses in ethnobotany: their relevance for ethnopharmacology and pharmacognosy in the context of historical data. Rev Bras Farmacogn. 2020;30:451–66.

[36] Reyes-García V, Benyei P, Aceituno-Mata L, Gras A, Molina M, Tardío J, Pardo-de-Santayana M. Documenting and protecting traditional knowledge in the era of open science: Insights from two Spanish initiatives. J Ethnopharmacol. 2021;278:114295. 34090912 10.1016/j.jep.2021.114295

[37] Berlin B, Breedlove DE, Raven PH. Folk taxonomies and biological classification. Science. 1966;154(3746):273–5. 17810308 10.1126/science.154.3746.273

[38] Berlin B, Kay P. Basic color terms: their universality and evolution. Berkley: University of California Press; 1969.

[39] Saunders B. Revisiting basic color terms. J R Anthropol Inst. 2000;6(1):81–99.

[40] Ulrich RS. View through a window may influence recovery from surgery. Science. 1984;224(4647):420–1. 6143402 10.1126/science.6143402

[41] Doran-Sherlock R, Devitt S, Sood P. An integrative review of the evidence for Shinrin-Yoku (Forest Bathing) in the management of depression and its potential clinical application in evidence-based osteopathy. J Bodyw Mov Ther. 2023;35:244–55. 37330777 10.1016/j.jbmt.2023.04.038

[42] Furuyashiki A, Tabuchi K, Norikoshi K, Kobayashi T, Oriyama S. A comparative study of the physiological and psychological effects of forest bathing (Shinrin-yoku) on working age people with and without depressive tendencies. Environ Health Prev Med. 2019;24(1):46. 31228960 10.1186/s12199-019-0800-1 PMC6589172

[43] Song I, Baek K, Kim C, Song C. Effects of nature sounds on the attention and physiological and psychological relaxation. Urban For Urban Green. 2023;86:127987.

[44] Briki W, Majed L. Adaptive effects of seeing green environment on psychophysiological parameters when walking or running. Front Psychol. 2019;10:252. 30809177 10.3389/fpsyg.2019.00252 PMC6379348

[45] Maffei ME, Gertsch J, Appendino G. Plant volatiles: production, function and pharmacology. Nat Prod Rep. 2011;28(8):1359–80. 21670801 10.1039/c1np00021g

[46] Gouk P. Musical healing in cultural contexts. London: Routledge; 2000.

[47] Chanda ML, Levitin DJ. The neurochemistry of music. Trends Cogn Sci. 2013;17(4):179–93. 23541122 10.1016/j.tics.2013.02.007

[48] Moreira SV, Justi FRDR, Moreira M. Can musical intervention improve memory in Alzheimer’s patients? Evidence from a systematic review. Dement Neuropsychol. 2018;12(2):133–42. 29988347 10.1590/1980-57642018dn12-020005 PMC6022981

[49] Moreno-Morales C, Calero R, Moreno-Morales P, Pintado C. Music therapy in the treatment of dementia: a systematic review and meta-analysis. Front Med (Lausanne). 2020;7:160. 32509790 10.3389/fmed.2020.00160 PMC7248378

[50] Shang A, Huwiler-Müntener K, Nartey L, Jüni P, Dörig S, Sterne JA, Pewsner D, Egger M. Are the clinical effects of homoeopathy placebo effects? Comparative study of placebo-controlled trials of homoeopathy and allopathy. Lancet. 2005;366(9487):726–32. 16125589 10.1016/S0140-6736(05)67177-2

[51] Singh S, Ernst E. Trick or treatment: the undeniable facts about alternative medicine. New York: WW Norton & Co Inc; 2009.

[52] Sorokowska A, Drechsler E, Karwowski M, Hummel T. Effects of olfactory training: a meta-analysis. Rhinology. 2017;55(1):17–26. 28040824 10.4193/Rhin16.195

[53] Speth MM, Singer-Cornelius T, Oberle M, Gengler I, Brockmeier SJ, Sedaghat AR. Mood, anxiety and olfactory dysfunction in COVID-19: evidence of central nervous system involvement? Laryngoscope. 2020;130(11):2520–5. 32617983 10.1002/lary.28964 PMC7361512

[54] Kohli P, Soler ZM, Nguyen SA, Muus JS, Schlosser RJ. The association between olfaction and depression: a systematic review. Chem Senses. 2016;41(6):479–86. 27170667 10.1093/chemse/bjw061 PMC4918728

[55] Perry N, Perry E. Aromatherapy in the management of psychiatric disorders: clinical and neuropharmacological perspectives. CNS Drugs. 2006;20:257–80. 16599645 10.2165/00023210-200620040-00001

[56] Trotter RT. Susto: the context of community morbidity patterns. Ethnology. 1982;1982(21):215–26.

[57] Hunn ES. A Zapotec natural history: trees, herbs, and flowers, birds, beasts, and bugs in the life of San Juan Gbëë. Tucson: University of Arizona Press; 2008. p. 2008.

[58] Geck MS, Cristians S, Berger-González M, Casu L, Heinrich M, Leonti M. Traditional herbal medicine in mesoamerica: toward its evidence base for improving universal health coverage. Front Pharmacol. 2020;11:1160. 32848768 10.3389/fphar.2020.01160 PMC7411306

[59] Casagrande DG. Human taste and cognition in tzeltal maya medicinal plant use. J Ecol Anthropol. 2000;4:57–69.

[60] Molares S, Ladio A. Chemosensory perception and medicinal plants for digestive ailments in a Mapuche community in NW Patagonia, Argentina. J Ethnopharmacol. 2009;123(3):397–406. 19501272 10.1016/j.jep.2009.03.033

[61] Dragos D, Gilca M. Taste of phytocompounds: a better predictor for ethnopharmacological activities of medicinal plants than the phytochemical class? J Ethnopharmacol. 2018;220:129–46. 29604378 10.1016/j.jep.2018.03.034

[62] Pieroni A, Morini G, Piochi M, Sulaiman N, Kalle R, Haq SM, Devecchi A, Franceschini C, Zocchi DM, Migliavada R, Prakofjewa J, Sartori M, Krigas N, Ahmad M, Torri L, Sõukand R. Bitter is better: wild greens used in the blue Zone of Ikaria, Greece. Nutrients. 2023;15(14):3242. 37513661 10.3390/nu15143242 PMC10385191

[63] Leonti M, Cabras S, Castellanos Nueda ME, Casu L. Food drugs as drivers of therapeutic knowledge and the role of chemosensory qualities. J Ethnopharmacol. 2024;28(328):118012. 10.1016/j.jep.2024.118012 38447614

[64] Mann RD. Modern drug use: an enquiry on historical principles. Lancaster: MTP Press Limited; 1984.

[65] Le Fanu J. The rise and fall of modern medicine. London: Hachette Digital; 2011. 10.1016/S0140-6736(05)75559-8 10465212

[66] Huxley A. Brave new world. London: Triad Grafton Books; 1932.

[67] Last J, Spasoff RA, Harris S. A dictionary of epidemiology. 4th ed. Oxford: Oxford University Press; 2001.

[68] Witt CM. Clinical research on traditional drugs and food items—the potential of comparative effectiveness research for interdisciplinary research. J Ethnopharmacol. 2013;147:254–8. 23458921 10.1016/j.jep.2013.02.024

[69] Gertsch J. How scientific is the science in ethnopharmacology? Historical perspectives and epistemological problems. J Ethnopharmacol. 2009;122(2):177–83. 19185054 10.1016/j.jep.2009.01.010

[70] Bruhn JG, Rivier L. Ethnopharmacology—a journal, a definition and a society. J Ethnopharmacol. 2019;242:112005. 31216432 10.1016/j.jep.2019.112005

[71] UN General Assembly. Universal declaration of human rights.: New York City: UN General Assembly; 1948.

[72] UN General Assembly. Transforming our world: the 2030 Agenda for sustainable development. New York City: UN General Assembly; 2015.

[73] Marshall SJ. Developing countries face double burden of disease. Bull World Health Organ. 2004;82(7):556. 15500291 PMC2622909

[74] Engels D, Zhou XN. Neglected tropical diseases: an effective global response to local poverty-related disease priorities. Infect Dis Poverty. 2020;9(1):10. 31987053 10.1186/s40249-020-0630-9 PMC6986060

[75] Odonne G, Musset L, Cropet C, Philogene B, Gaillet M, Tareau MA, Douine M, Michaud C, Davy D, Epelboin L, Lazrek Y, Brousse P, Travers P, Djossou F, Mosnier E. When local phytotherapies meet biomedicine. Cross-sectional study of knowledge and intercultural practices against malaria in Eastern French Guiana. J Ethnopharmacol. 2021;279:114384. 34217796 10.1016/j.jep.2021.114384

[76] Houël E, Ginouves M, Azas N, Bourreau E, Eparvier V, Hutter S, Knittel-Obrecht A, Jahn-Oyac A, Prévot G, Villa P, Vonthron-Sénécheau C, Odonne G. Treating leishmaniasis in Amazonia, part 2: multi-target evaluation of widely used plants to understand medicinal practices. J Ethnopharmacol. 2022;289:115054. 35131338 10.1016/j.jep.2022.115054

[77] Salm A, Krishnan SR, Collu M, Danton O, Hamburger M, Leonti M, Almanza G, Gertsch J. Phylobioactive hotspots in plant resources used to treat Chagas disease. iScience. 2021;24(4):102310. 33870129 10.1016/j.isci.2021.102310 PMC8040286

[78] Elmi A, Said Mohamed A, Mérito A, Charneau S, Amina M, Grellier P, Bouachrine M, Lawson AM, Abdoul-Latif FM, Kordofani MAY. The ethnopharmacological study of plant drugs used traditionally in Djibouti for malaria treatment. J Ethnopharmacol. 2024;325:117839. 38310984 10.1016/j.jep.2024.117839

[79] Feasey N, Wansbrough-Jones M, Mabey DC, Solomon AW. Neglected tropical diseases. Br Med Bull. 2010;93:179–200. 20007668 10.1093/bmb/ldp046

[80] Willcox ML, Graz B, Falquet J, Diakite C, Giani S, Diallo D. A “reverse pharmacology” approach for developing an anti-malarial phytomedicine. Malar J. 2011;10(Suppl 1):8. 21411019 10.1186/1475-2875-10-S1-S8 PMC3059466

[81] Mayer M, Vogl CR, Amorena M, Hamburger M, Walkenhorst M. Treatment of organic livestock with medicinal plants: a systematic review of European ethnoveterinary research. Forsch Komplementmed. 2014;21(6):375–86. 25592949 10.1159/000370216

[82] Ayrle H, Nathues H, Bieber A, Durrer M, Quander N, Mevissen M, Walkenhorst M. Placebo-controlled study on the effects of oral administration of *Allium sativum* L. in postweaning piglets. Vet Rec. 2019;184(10):316. 30777882 10.1136/vr.105131

[83] Still J. Use of animal products in traditional Chinese medicine: environmental impact and health hazards. Complement Ther Med. 2003;11(2):118–22. 12801499 10.1016/s0965-2299(03)00055-4

[84] Starr C, Nekaris KAI, Streicher U, Leung L. Traditional use of slow lorises *Nycticebus bengalensis* and *N. pygmaeus* in Cambodia: an impediment to their conservation. Endange Spec Res. 2010;12:17–23.

[85] Baker SE, Cain R, van Kesteren F, Zommers ZA, D’Cruze N, Macdonald DW. Rough trade: animal welfare in the global wildlife trade. Bioscience. 2013;63:928–38.

[86] Moorhouse TP, Coals PGR, D’Cruze NC, Macdonald DW. Reduce or redirect? Which social marketing interventions could influence demand for traditional medicines. Biol Cons. 2020;242:108391.

[87] Moorhouse TP, D’Cruze NC, Sun E, Elwin A, Macdonald DW. What are TCM doctors’ attitudes towards replacing animal-origin medicinal materials with plant-origin alternatives? Glob Ecol Conserv. 2020;34:e02045.

